# Supplementary material for: Good conduct makes your face attractive: The effect of personality perception on facial attractiveness judgments
Source: PLoS One. 2023 Feb 13;18(2):e0281758. doi: 10.1371/journal.pone.0281758 (PMC9925008; doi:10.1371/journal.pone.0281758)

S1 Figure. Schematic illustration of the mediation analysis on Experiment 2 (N = 457). The honesty item of the personality ratings was used as the independent variable (perceived honesty), and the likability item of the personality ratings was used as the mediator (perceived likability). The attractiveness ratings of the face images were significantly mediated by the perceived likability. Standardized coefficients are in the parentheses.

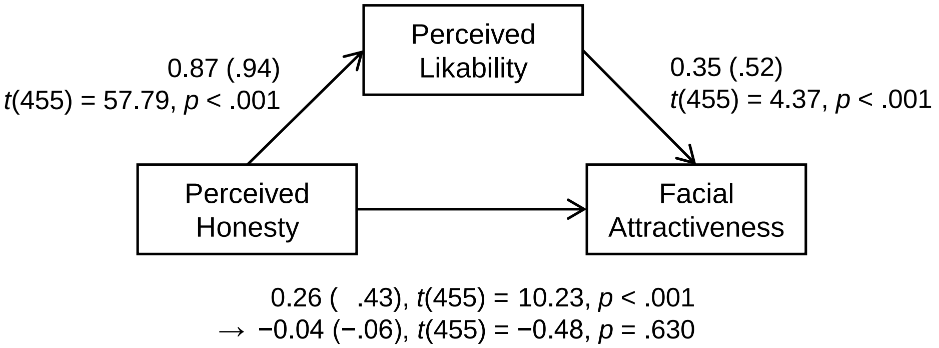

Supplement: S1 Fig — (PDF) [file pone.0281758.s001.pdf]
