# Supplementary material for: Good conduct makes your face attractive: The effect of personality perception on facial attractiveness judgments
Source: PLoS One. 2023 Feb 13;18(2):e0281758. doi: 10.1371/journal.pone.0281758 (PMC9925008; doi:10.1371/journal.pone.0281758)
Supplement: S1 Table — (PDF) [file pone.0281758.s002.pdf]

S1 Table. Results of Experiment 1.

Rating item	Low pre-rated facial attractiveness		High pre-rated facial attractiveness		ANOVA *p*		
	Low honesty (*n* = 17)	High honesty (*n* = 15)	Low honesty (*n* = 17)	High honesty (*n* = 16)	Honesty	Pre-rated facial attractiveness	Honesty×Pre-rated facial attractiveness
Personality rating							
Unintelligent–Intelligent	6.00 (2.26)	7.37 (1.14)	6.59 (1.42)	7.69 (0.79)	.002	.235	.725
Dependent–Independent	3.29 (1.93)	5.27 (2.15)	4.24 (1.56)	6.56 (1.46)	< .001	.015	.691
Dishonest–Honest	2.65 (2.09)	8.13 (1.19)	2.76 (2.08)	8.06 (1.00)	< .001	.956	.823
Calm–Anxious	3.59 (2.15)	5.36 (2.34)	4.29 (1.76)	4.62 (2.42)	.059	.981	.192
Unambitious–Ambitious	4.76 (2.02)	2.86 (1.35)	4.12 (1.62)	3.50 (1.79)	.005	.996	.141
Unsociable–Sociable	3.12 (1.69)	6.13 (1.68)	4.29 (1.83)	5.31 (1.25)	< .001	.663	.017
Dislike–Like	2.71 (1.99)	7.67 (1.29)	2.88 (2.09)	7.19 (1.22)	< .001	.723	.444
Physical rating							
Unattractive–Attractive	3.88 (1.80)	5.33 (1.80)	5.47 (2.24)	6.75 (1.44)	.004	.002	.852
Mature faced–Baby faced	4.00 (2.10)	3.71 (1.77)	3.21 (1.26)	3.12 (1.02)	.650	.090	.800
Masculine–Feminine	4.50 (2.39)	4.87 (2.00)	5.53 (2.48)	5.56 (2.42)	.734	.146	.777
Mean–Kind	5.50 (1.97)	7.13 (1.25)	5.21 (1.78)	7.00 (1.21)	< .001	.593	.840
Poor fitness–Good fitness	5.59 (2.45)	5.53 (1.88)	5.00 (2.06)	6.06 (1.12)	.303	.952	.254
Poor health–Good health	7.00 (1.62)	7.60 (1.45)	6.82 (1.29)	7.12 (1.02)	.189	.341	.662
Short–Tall	6.15 (2.03)	5.73 (1.79)	6.88 (1.73)	6.50 (1.79)	.387	.106	.973
Underweight–Overweight	4.85 (1.80)	4.93 (1.67)	4.00 (2.09)	4.16 (1.23)	.785	.063	.930
Small eyes–Large eyes	5.31 (1.74)	5.50 (1.09)	7.24 (0.90)	7.25 (1.00)	.743	< .001	.779
Close eyes–Spaced eyes	4.82 (1.98)	4.73 (0.88)	5.24 (1.56)	5.50 (1.32)	.816	.121	.637
Almond eyes–Round eyes	4.53 (2.29)	4.43 (1.22)	6.35 (1.11)	6.09 (1.77)	.672	< .001	.852
Small nose–Large nose	6.38 (1.54)	6.07 (1.14)	6.00 (1.54)	5.25 (1.34)	.140	.095	.538
Coarse hair–Fine hair	4.00 (2.76)	3.67 (1.72)	3.82 (1.67)	4.56 (1.86)	.694	.486	.300
Thin lips–Full lips	6.47 (1.86)	5.63 (1.23)	4.82 (1.63)	4.81 (1.56)	.292	.003	.305
Narrow chin–Wide chin	4.88 (2.22)	5.67 (1.76)	4.71 (1.65)	5.19 (1.72)	.174	.468	.739
Rough skin–Smooth skin	5.93 (1.58)	6.36 (0.93)	6.65 (1.73)	6.69 (1.35)	.531	.162	.605
Stout neck–Graceful neck	4.18 (2.19)	4.20 (2.40)	4.47 (1.87)	4.44 (2.10)	.993	.619	.958
Angular face–Round face	4.06 (1.78)	3.40 (1.72)	4.24 (1.60)	5.31 (0.87)	.587	.008	.027
Low cheekbones–High cheekbones	4.59 (1.80)	4.79 (1.76)	5.18 (1.59)	5.56 (1.46)	.486	.106	.821
Asymmetrical–Symmetrical	5.24 (2.28)	5.47 (2.10)	5.35 (2.12)	5.06 (1.88)	.955	.785	.619

Note. Standard deviations are presented in parentheses.
