# Supplementary material for: Good conduct makes your face attractive: The effect of personality perception on facial attractiveness judgments
Source: PLoS One. 2023 Feb 13;18(2):e0281758. doi: 10.1371/journal.pone.0281758 (PMC9925008; doi:10.1371/journal.pone.0281758)
Supplement: S2 Table — (PDF) [file pone.0281758.s003.pdf]

S2 Table. Results of Experiment 2.

Rating item	Low shirt attractiveness				High shirt attractiveness				ANOVA *p*		
	Low honesty (*n* = 110)		High honesty (*n* = 105)		Low honesty (*n* = 133)		High honesty (*n* = 109)		Honesty	Shirt attractiveness	Honesty×Shirt attractiveness
Personality rating											
Unintelligent–Intelligent	58.1	(22.6)	80.8	(14.5)	57.6	(24.0)	82.0	(13.9)	< .001	.853	.832
Dependent–Independent	39.7	(23.3)	57.1	(21.2)	37.2	(21.9)	61.8	(16.5)	< .001	.441	.075
Dishonest–Honest	18.4	(22.2)	89.9	(13.2)	19.7	(22.7)	89.1	(13.1)	< .001	.906	.439
Calm–Anxious	45.7	(20.2)	22.7	(15.0)	45.2	(19.5)	22.2	(14.8)	< .001	.952	.668
Unambitious–Ambitious	48.4	(21.8)	34.9	(18.9)	49.0	(23.7)	35.3	(17.6)	< .001	.621	.931
Unsociable–Sociable	40.7	(17.6)	61.4	(14.7)	41.4	(19.2)	60.8	(13.6)	< .001	.912	.604
Dislike–Like	20.0	(21.6)	84.2	(13.6)	21.3	(22.2)	83.8	(16.1)	< .001	.911	.425
Physical rating											
Unattractive–Attractive	40.1	(21.0)	61.0	(21.8)	43.5	(25.0)	62.1	(20.5)	< .001	.285	.631
Masculine–Feminine	37.9	(23.7)	46.0	(28.1)	40.1	(26.7)	46.8	(29.3)	< .001	.695	.333
Mean–Kind	42.6	(21.8)	65.2	(22.0)	42.4	(21.9)	64.0	(21.3)	< .001	.625	.866
Poor health–Good health	54.6	(23.2)	69.7	(23.4)	65.3	(21.0)	72.1	(20.8)	< .001	.003	.072
Small eyes–Large eyes	64.4	(17.1)	67.0	(19.8)	64.2	(19.0)	67.5	(19.3)	.201	.826	.999
Coarse hair–Fine hair	57.5	(22.0)	55.5	(23.0)	57.3	(24.5)	54.0	(22.5)	.246	.962	.697
Stout neck–Graceful neck	35.5	(22.5)	33.8	(22.2)	37.0	(23.9)	37.8	(23.5)	.712	.167	.451
Angular face–Round face	44.6	(24.5)	49.7	(25.3)	46.4	(26.7)	48.2	(26.2)	.255	.653	.176

Note. Standard deviations are presented in parentheses. Four-factoral ANOVA (honesty, shirt attractiveness, target gender, participant gender) was applied to each item.
