# Supplementary material for: Good conduct makes your face attractive: The effect of personality perception on facial attractiveness judgments
Source: PLoS One. 2023 Feb 13;18(2):e0281758. doi: 10.1371/journal.pone.0281758 (PMC9925008; doi:10.1371/journal.pone.0281758)
Supplement: S3 Table — (PDF) [file pone.0281758.s004.pdf]

S3 Table. Results of Experiment 3.

Rating item	Low creativity		High creativity		ANOVA *p*		
	Low aggressiveness (*n* = 31)	High aggressiveness (*n* = 30)	Low aggressiveness (*n* = 28)	High aggressiveness (*n* = 28)	Aggressiveness	Creativity	Aggressiveness ×Creativity
Personality rating							
Unintelligent–Intelligent	5.94 (1.50)	4.20 (1.58)	7.71 (1.44)	6.07 (1.46)	< .001	< .001	.868
Dependent–Independent	7.13 (1.34)	5.67 (1.83)	7.96 (1.20)	6.43 (1.79)	< .001	.007	.899
Dishonest–Honest	7.74 (1.29)	4.13 (1.63)	8.18 (0.98)	4.75 (1.71)	< .001	.050	.735
Calm–Anxious	2.06 (0.96)	6.87 (1.53)	2.36 (1.50)	6.96 (1.23)	< .001	.426	.691
Unaggressive–Aggressive	2.06 (0.89)	7.20 (1.58)	1.89 (0.96)	7.57 (1.03)	< .001	.641	.206
Unambitious–Ambitious	3.42 (1.95)	3.77 (1.77)	5.04 (1.99)	7.04 (1.37)	< .001	< .001	.014
Unsociable–Sociable	6.42 (1.80)	4.73 (1.95)	7.46 (1.73)	6.57 (1.73)	< .001	< .001	.238
Uncreative–Creative	2.71 (1.04)	2.40 (1.16)	7.68 (1.31)	7.96 (1.10)	.955	< .001	.166
Dislike–Like	6.77 (1.45)	4.27 (1.55)	7.32 (1.91)	4.93 (2.19)	< .001	.070	.863
Physical rating							
Unattractive–Attractive	5.32 (1.62)	4.30 (1.73)	5.04 (1.82)	4.86 (1.84)	.066	.677	.195
Mature faced–Baby faced	4.26 (2.03)	4.00 (1.88)	3.32 (1.76)	3.79 (1.75)	.766	.098	.298
Masculine–Feminine	4.81 (2.65)	3.93 (1.82)	4.29 (2.43)	3.86 (2.34)	.134	.490	.607
Mean–Kind	5.90 (1.89)	5.43 (2.01)	6.04 (1.57)	5.14 (1.76)	.046	.815	.532
Poor fitness–Good fitness	5.52 (2.25)	6.23 (1.91)	5.93 (1.92)	6.46 (1.88)	.093	.387	.807
Poor health–Good health	6.58 (1.41)	6.17 (1.88)	6.57 (1.79)	6.46 (2.03)	.432	.663	.643
Short–Tall	5.87 (2.00)	5.80 (2.27)	6.07 (2.05)	5.25 (2.35)	.269	.664	.352
Underweight–Overweight	5.71 (1.70)	5.70 (1.70)	5.43 (2.04)	5.43 (1.83)	.989	.414	.989
Small eyes–Large eyes	6.35 (1.89)	6.47 (1.55)	6.54 (1.64)	6.25 (1.76)	.785	.955	.533
Coarse hair–Fine hair	4.26 (2.27)	4.50 (2.13)	4.29 (1.96)	5.54 (2.17)	.062	.182	.205
Stout neck–Graceful neck	3.55 (1.63)	3.17 (1.21)	3.32 (1.68)	2.82 (1.06)	.096	.279	.822
Angular face–Round face	5.35 (2.26)	4.77 (1.83)	4.96 (2.12)	4.54 (2.19)	.194	.427	.838

Note. Standard deviations are presented in parentheses.
