# Supplementary material for: Good conduct makes your face attractive: The effect of personality perception on facial attractiveness judgments
Source: PLoS One. 2023 Feb 13;18(2):e0281758. doi: 10.1371/journal.pone.0281758 (PMC9925008; doi:10.1371/journal.pone.0281758)
Supplement: S4 Table — (PDF) [file pone.0281758.s005.pdf]

S4 Table. Results of Experiment 4.

Rating item	Relevant				Irrelevant				ANOVA *p*		
	Low honesty (*n* = 33)		High honesty (*n* = 33)		Low honesty (*n* = 32)		High honesty (*n* = 32)		Honesty	Relevance	Honesty ×Relevance
Personality rating											
Unintelligent–Intelligent	6.61	(1.97)	7.52	(1.15)	5.31	(2.13)	7.59	(1.01)	< .001	.037	.019
Dependent–Independent	3.52	(1.56)	5.21	(1.96)	3.41	(1.41)	5.88	(1.83)	< .001	.357	.200
Dishonest–Honest	2.03	(1.19)	7.73	(1.59)	2.28	(1.35)	8.12	(1.13)	< .001	.166	.753
Calm–Anxious	4.70	(2.04)	2.55	(1.23)	4.06	(2.02)	2.88	(1.10)	< .001	.600	.099
Unambitious–Ambitious	4.39	(2.18)	3.09	(1.38)	5.56	(1.83)	2.84	(1.14)	< .001	.121	.018
Unsociable–Sociable	3.61	(1.39)	5.91	(1.49)	4.28	(1.57)	5.88	(1.31)	< .001	.208	.164
Dislike–Like	2.24	(1.25)	7.42	(1.68)	2.72	(1.46)	7.22	(0.97)	< .001	.574	.158
Physical rating											
Unattractive–Attractive	4.58	(1.70)	4.97	(1.76)	4.84	(1.95)	5.03	(1.56)	.344	.592	.737
Masculine–Feminine	4.39	(2.34)	4.24	(2.36)	4.31	(2.46)	4.22	(2.37)	.770	.900	.945
Mean–Kind	4.39	(1.77)	5.79	(1.63)	5.34	(1.45)	5.53	(1.78)	.008	.237	.041
Poor health–Good health	6.03	(1.86)	6.33	(1.65)	6.59	(1.54)	6.12	(1.74)	.782	.553	.199
Small eyes–Large eyes	6.09	(1.47)	6.24	(1.50)	6.34	(1.68)	6.03	(1.47)	.765	.938	.389
Coarse hair–Fine hair	4.15	(2.22)	5.67	(1.85)	4.44	(2.00)	4.59	(1.86)	.018	.262	.054
Stout neck–Graceful neck	4.30	(1.78)	3.24	(1.54)	3.38	(1.60)	3.53	(1.41)	.108	.254	.031
Angular face–Round face	4.67	(1.65)	4.33	(1.67)	3.88	(2.01)	4.38	(1.96)	.796	.245	.197
Mood rating											
PA	2.89	(1.28)	3.14	(1.43)	2.87	(1.38)	2.90	(1.44)	.553	.586	.642
NA	1.88	(1.23)	1.49	(1.15)	2.04	(1.50)	1.76	(1.13)	.128	.345	.799

Note. Standard deviations are presented in parentheses. PA = positive affect, NA = negative affect.
