# Supplementary material for: Good conduct makes your face attractive: The effect of personality perception on facial attractiveness judgments
Source: PLoS One. 2023 Feb 13;18(2):e0281758. doi: 10.1371/journal.pone.0281758 (PMC9925008; doi:10.1371/journal.pone.0281758)
Supplement: S1 Appendix — (PDF) [file pone.0281758.s006.pdf]

S1 Appendix. Experiment 3 personality descriptions.

The description of male target with low aggressiveness and low creativity was as follows (translated in English here).

> Shōta is a university student. He manages his studies, part-time job, and club activities well, and he enjoys his university life. He has not lost any credits and is making good progress with his thesis work.
>
> Shōta works a part-time job at a convenience store. He works hard and is very attentive to customers. A friend working with him has said that he does not seem annoyed with any customer and that he is polite and smiles. Even in daily life, Shōta rarely hits things even if he gets frustrated.
>
> Shōta is also very active in his club. However, when the club holds events, he rarely comes up with new ideas that no one else has thought of. When a friend invited him to visit an art museum, he didn't seem very interested in looking at paintings. In fact, he never paints and claims he is not good at creating his own artwork.

The personality description of female target with high aggressiveness and high creativity was as follows (translated in English here).

> Misaki is a university student. She manages her studies, part-time job, and club activities well, and she enjoys her university life. She has not lost any credits and is making good progress with her thesis work.
>
> Misaki works a part-time job at a convenience store. She works hard but sometimes has problems with customers. A friend working with her has heard her having verbal fights and yelling at customers many times. Misaki sometimes hits things or kicks chairs when she gets frustrated in daily life.
>
> Misaki is also very active in her club. When the club holds events, she comes up with a lot of new interesting ideas that no one else has thought of. She also likes to see paintings and other artwork and frequently visits art museums with her friends. In fact, she paints in oil and submits her paintings to competitions every year.
