# Supplementary material for: Good conduct makes your face attractive: The effect of personality perception on facial attractiveness judgments
Source: PLoS One. 2023 Feb 13;18(2):e0281758. doi: 10.1371/journal.pone.0281758 (PMC9925008; doi:10.1371/journal.pone.0281758)
Supplement: Attachment — Submitted filename: ResponseToReviewers.docx [file pone.0281758.s007.docx]

フォームの始まり

フォームの終わり

フォームの始まり

フォームの終わり

PONE-D-22-24839 “Good conduct makes your face attractive: The effect of personality perception on facial attractiveness judgments”

Responses to “Journal Requirements” comments:

(Page numbers and line numbers in the replies are those of the revised paper)

1. Please ensure that your manuscript meets PLOS ONE's style requirements, including those for file naming. The PLOS ONE style templates can be found at

https://journals.plos.org/plosone/s/file?id=wjVg/PLOSOne_formatting_sample_main_body.pdf and

https://journals.plos.org/plosone/s/file?id=ba62/PLOSOne_formatting_sample_title_authors_affiliations.pdf.

We checked the materials of the revised paper so that they adhere to the requirements. The figure image files were verified by PACE.

2. Please provide additional details regarding participant consent. In the ethics statement in the Methods and online submission information, please ensure that you have specified what type you obtained (for instance, written or verbal, and if verbal, how it was documented and witnessed). If your study included minors, state whether you obtained consent from parents or guardians. If the need for consent was waived by the ethics committee, please include this information.

We obtained the participants’ consents to participante in the experiments by confirming that they marked the “I agree to participate” box on the cover page of our online forms. See the ethics statements in the manuscript (p. 6, line 29). All experiments were conducted online. This procedure was approved in advance by the IRB. All participants were adult.

3. Thank you for stating the following in the Competing Interests section:

"RN received a grant from the KOSE Kosmetology Research Foundation. MG declares no competing interests."

Please confirm that this does not alter your adherence to all PLOS ONE policies on sharing data and materials, by including the following statement: "This does not alter our adherence to PLOS ONE policies on sharing data and materials.” (as detailed online in our guide for authors http://journals.plos.org/plosone/s/competing-interests).  If there are restrictions on sharing of data and/or materials, please state these. Please note that we cannot proceed with consideration of your article until this information has been declared.

Please include your updated Competing Interests statement in your cover letter; we will change the online submission form on your behalf.

We corrected the Competing Interests statements as suggested.

4. Your ethics statement should only appear in the Methods section of your manuscript. If your ethics statement is written in any section besides the Methods, please move it to the Methods section and delete it from any other section. Please ensure that your ethics statement is included in your manuscript, as the ethics statement entered into the online submission form will not be published alongside your manuscript.

The ethical statements were moved to the Method section of the revised manuscript (pp. 6–7).

5. We note that Figure 1 includes images of a participants in the study. 
As per the PLOS ONE policy (http://journals.plos.org/plosone/s/submission-guidelines#loc-human-subjects-research) on papers that include identifying, or potentially identifying, information, the individual(s) or parent(s)/guardian(s) must be informed of the terms of the PLOS open-access (CC-BY) license and provide specific permission for publication of these details under the terms of this license. Please download the Consent Form for Publication in a PLOS Journal (http://journals.plos.org/plosone/s/file?id=8ce6/plos-consent-form-english.pdf). The signed consent form should not be submitted with the manuscript, but should be securely filed in the individual's case notes. Please amend the methods section and ethics statement of the manuscript to explicitly state that the patient/participant has provided consent for publication: “The individual in this manuscript has given written informed consent (as outlined in PLOS consent form) to publish these case details”.

If you are unable to obtain consent from the subject of the photograph, you will need to remove the figure and any other textual identifying information or case descriptions for this individual.

All the face images in Fig 1 were adopted from publicly available stock photography (https://www.photo-ac.com/). Please see the ethical statements in the manuscript (pp. 6–7). We did not take any picture of our participants.

PONE-D-22-24839 “Good conduct makes your face attractive: The effect of personality perception on facial attractiveness judgments”

Responses to the comments by Reviewer #1

(Page numbers and line numbers in the replies are those of the revised paper)

1) The manuscript was generally well written. I note a few confusing statements below. My greatest concern is how Experiment 4 produces inconclusive results but the authors interpret them to mean that positive or negative affect is not involved in the process. I believe the contribution would be more scientifically clear if Exp. 4 were removed or if the researchers conducted another experiment to clarify the results of Exp. 4. Below I give my comments in the order in which they appeared in the manuscript.

We realize that the results of Experiment 4 are somewhat ambiguous and difficult to interpret. Nevertheless we believe that reporting Exerpiment 4 is beneficial for readers because i) there has been no study examing the potential effect of moods on the honesty premium effect, ii) Experiment 4 was pre-registered and removing it from the paper because of replication failure would reinforce the publication bias, and iii) there were multiple pieces of evidence (though indirect) suggesting that the mood hypothesis was relatively unlikely. Some of them were found by additional analyses, including those suggested by the reviewers.

The first piece of evidence against the mood hypothesis was the significant relevance×honesty interactions in some items of the physical ratings (p. 18, line 8–11). Two physical rating items were modified by the honesty manipulation only for the relevant target faces. In the irrelevant condition, there was no physical rating item influenced by honesty. This pattern of results suggests that the effect of perceiving honest personalitiy on the physical ratings was not mediated by moods.

Second, we conducted an additonal analysis on the potential effect of moods on the attractiveness ratings. We examined the multiple regression model to fit the attractiveness ratings by two independent variables of PA socre and NA score (see p. 18, line 29–p. 19, line 2). The coefficients of neither variable were statistically significant. Further, the Bayes factor showed that the null model (i.e., intercept only) was favored against the regression model by PA/NA scores (*BF*_01_ = 3.67). These results suggested that the participants’ moods did not relate to the perceived facial attractiveness.

Third, we found that the mood ratings were (at least partly) successful. Following the reviewer’s suggestion, we examined the results of each mood rating item. One NA item (“unpleasant”) was significantly influenced by the honesty manipulation. Importantly, a regression analysis showed that the “unpleasant” ratings did not correlate with the attractiveness ratings (*b* = −0.03, *p* = .774; *BF*_01_ = 5.14). This was reported in the revised paper (p.19, line 3).

Given these data, we think that Experiment 4 provides moderate evidance that the mood hypothesis was not very likely. On the other hand, the Paunonen’s (2006) explanation that the honesty effect on the physical attractiveness judgments is mediated by general likability seemed more likely, as we also found that the significant mediation effect of likability (see our last reply below).

Nevertheless, these findings were indirect evidence and we could not draw a definitive conlusion on the mood hypothesis. We pointed this issue in the revised Discussion section of Experiment 4: “Despite replication failure, the pattern of the results was generally inconsistent with the mood hypothesis. First, the participants’ mood ratings did not correlate with the facial attractiveness ratings. Second, a significant effect of honesty on the physical ratings of target faces was found only in the relevant condition. Nevertheless, we could not draw definite conclusions on the mood hypothesis because of the replication failure of the honesty premium effect. Further study is warranted.” (p. 19, line 15–20)

2) In the abstract, the authors write "more or less concerned." This is unclear. Writing, "Most people are at least somewhat concerned..." would be more clear.

Corrected as suggested (p. 3, line 5).

3) In the abstract, the authors write "personal traits" when they mean "personality traits" (I believe).

Corrected as suggested (p. 3, line 7).

4) In the abstract, the word "influence" is used, but it would be more accurate to write "correlated." This is because "influence" implies causality but most of the research is correlational (it is difficult to randomly assign folks to physical attractiveness levels).

We revised the paper throughout so that “correlate” and “relate” were used to refer to correlational data. We also took care to distinguish “(physical) attractiveness” and “attractiveness judgments/ratings” in our use of the terms. Thank you for the suggestion.

5) Page 4, line 17 -- "yielded" is unclear; "gave" would be more accurate.

Corrected as suggested (p. 4, line 21).

6) In Study 1, the authors explain the sample size by writing that it was determined following the previous study. This is not clear. Simply using the same sample size as past is not good practice. Power calculations are needed. This same comment applies to all experiments.

Yes, the sample size of Experiment 1 was determined by the conventional practice to follow the previous study. A priori sample-size estimation was not done. We realize that this is not good. The Experiment 1 result was justified by the post hoc power analysis. The achieved power for the effect of honesty was acceptable (0.856). We reported this result in the revised paper (p. 9, line 2).

For Experiment 2, we added the following sentences to the revised Method secion: “We recruited 480 participants through a crowdsourcing platform (crowdworks.jp). Since we assumed that crowdworkers would yield larger variance and more invalid responses than the student sample of Experiment 1, we planned 30 samples per condition” (p. 10, line 8–10). This was not based on power calculations. However, the achieved power was quite high (≃1) for the significant effect of honesty on attractiveness (p. 11, line 21).

The sample size calculation for Experiment 3 was reported in p. 13, line 9.

In Experiment 4, we had conducted a priori sample-size estimation. The revised Participants section reported the calculation: “A priori estimation of required sample size was 128 under the assumption of medium effect size (f = 0.25) of the two-way interaction (which was the main hypothesis of this experiment) with α = 0.05 and power = 0.8” (p. 16, line 26–28).

7) Page 7, line 5 -- "with the appearance of 20s" is unclear. Do you mean that the photo was shown for 20s? Just state it more directly, if so.

It was not about duration but about the apparent age of the faces. We corrected the sentence as follows: “Each image showed a face looking about 20–30 years old from the shoulders up” (p. 7, line 7–8).

8) Page 8, line 27 -- "seemed equal" -- At several places in the results, the authors want to claim that they proved the null hypothesis. This is incorrect. Bayes or some other statistics are needed to do this. See Aczel, B., Palfi, B., Szollosi, A., Kovacs, M., Szaszi, B., Szecsi, P., ... & Wagenmakers, E. J. (2018). Quantifying support for the null hypothesis in psychology: An empirical investigation. Advances in Methods and Practices in Psychological Science, 1(3), 357-366.). To claim support for a null hypothesis, Bayes statistics need to be used (jamovi and JASP are free software that can easily be used to calculate Bayes factors).

Thank you for the suggetion. We conducted some Bayes factor analyses using JASP and the BayesFactor package for R. We reported the results in the revised manuscript.

In Experiment 1, the null hypothesis for the honesty×pre-rated attractiveness interaction was not strongly supported: “A Bayes factor analysis showed that a linear model with the two fixed effects (honesty and pre-rated attractiveness) but without the interaction effect was favored against the full model by *BF* = 2.96, which was weak evidence.” (p. 9, line 4–6)

In Experiment 2, the null effects of participant gender, stimulus gender, and shirt attractiveness were supported. “Bayes factor analyses showed that the linear model with the fixed effect of honesty was more likely compared to the models with honesty×participant gender, honesty×target gender, or honesty×shirt attractiveness (*BF*s = 16.30, 5.01, and 33.24, respectively).” (p. 12, line 10–14)

In Experiment 3, the null effect of creativity was supported. “The absence of a creativity effect was supported by the moderate Bayes factor favoring the null hypothesis (*BF*_01_ = 4.75).” (p. 14, line 25–27)

In Experiment 4, the null effect of moods on the attractiveness ratings was confirmed. “A multiple linear regression model to fit the rated attractiveness with the independent variables of PA and NA scores found that neither of the regression coefficients was statistically significant (*b* = 0.24 and 0.11, respectively; *p*s > .05). Against this model, the null model was more likely (*BF*_01_ = 3.67). Likewise, the NA item “unpleasant” did not account for the attractiveness ratings (*b* = −0.03, *p* = .774; *BF*_01_ = 5.14).” (p. 18, line 29–p. 19, line 4)

9) Page 10, line 26-27: it is not clear why the sample size was too small to examine participant gender. This is also confusing because gender is tested in the middle of page 11.

This one participant reported “non-binary” for the gender identity question. If we were to incorporate this category into the analysis of the effect of participant gender, the sample size for the category (namely, 1) was too small. To clarify this, we revised the sentence as followed: “we excluded data from one participant who reported a non-binary gender identity because the sample size for the category (i.e., 1) was too small for statistical analysis of the effect of participant gender.” (p. 11, line 5–7)

10) Experiment 3 nicely reported a priori sample size determination methods.

(No response)

11) In Exp. 3, target creativity and aggressiveness were crossed in the manipulation, so that a target could be high creative and high aggressive. Why was this done? I suspect it complicates the results and probably led to the weaker results and lack of an effect in female targets (the odd, almost interactive effect in female targets suggests this). This should be discussed.

Although we did not have any working hypothesis regarding the aggressiveness×creativity interaction, we adopted the orthogonal design following the Paunonen’s (2006) study. Paunonen (2006) reported an unexpected interaction effect of independence and intelligence.

As the reviewer pointed out, out results showed a (non-significant) pattern of aggressiveness×creativity interaction for the female targets. Currently we do not have a clear interpretation on this pattern. Since the analysis on target gender was an ad hoc analysis, the sample size or the number of stimulus faces might not enough for the analysis. This issue may be examined in future research. We discussed this issue in the revised paper (p, 15, line 8–11).

12) Report standard deviations with each mean.

In the revised paper we reported SDs.

13) In Exp. 4, the manipulation of relevance is a good way to test the mood explanation. The measurement of mood is not. The mood ratings were made by participants after all other measures, rendering an insensitive measure even more insensitive. Also, the instructions given to participants for the mood ratings are not provided. They should be.

To avoild any influences of mood ratings on the face attractiveness ratings, we placed the mood ratings after the physical ratings. This was because the primary purpose of Experiment 4 was to test the relevance×honesty interaction. We had expected to show that the honesty effect would be present for the relevant targets but not for the irrelevant targets, so the replication of the honesty effect was critical. If the mood ratings were conducted before the physical ratings and the honsety effect was not replicated, the replication failure might be attributable to the presence of the mood ratings. Therefore we placed the mood ratings at the end in order to make the procedure of the relevant condition identical to the previous experiments. We designed the mood ratings as only a secondary tool to test the mood hypothesis.

We also found that the mood ratings were (at least partially) successful, as one item (“unpleasant”) was significantly modified by the honesty manipulation (see the next reply).

The instruction to the participants for the mood ratings was included in the revised Method section (“how well do the following words fit your current mood?” p. 17, line 13).

14) What happens when you test each mood word, as was done for the attractiveness ratings? The NA results are close to significant.

We applied the same ANOVA (honesty×relevance) to each of four PA items and five NA items. Only one item showed a significant effect: the “unpreasant” ratings were significantly increased by the dishonest target. However, the “unpreasant” ratings did not correlate with the facial attractiveness ratings, which was inconsistent with the mood hypothesis. These results were described in the Results section of the revised paper (p. 18, line 26–28).

15) In the end, Exp. 4 failed to replicate the honesty effect and the experiment does not aid in interpreting the effects. There are many possible reasons for the failure to replicate.

As the reviewer stated, at this time we cannot draw a definitive conclusion on the absence of the honesty effect in Experiment 4. We assume that motivational factors of the participants have some effect (see the Discussion section, p. 19, line 7–14). Despite the replication failure, we believe that it would be beneficial for future research to report Experiment 4, as we first experimentally examined the mood hypothesis.

16) In the discussion, mediation (via likability) is mentioned, but no formal statistical mediation is tested. Inclusion of these analyses would benefit the interpretation of the results.

We conducted a mediation analysis on the data of Experiment 2, which had a large sample (*N* = 457). By controlling the mediation effect by likability, the path from honesty to facial attractiveness lost its statistical significance.

This reults was reported in the Supplementary Fig S1 and in the revised General Discussion: “The mediation effect of likability was further confirmed by a mediation analysis on Experiment 2, which had a large sample. A significant direct effect from the rated honesty to the rated attractiveness (*b* = 0.26, *p* < .001) became non-significant (*b* = −0.04, *p* = .63) by controlling the rated likability as a mediator. The indirect effect mediated by the rated likability was statistically significant (Sobel’s test, *z* = 4.35, *p* < .001; see also S1 Fig).” (p. 20, line 27–p.21, line 2)

PONE-D-22-24839 “Good conduct makes your face attractive: The effect of personality perception on facial attractiveness judgments”

Responses to the comments by Reviewer #2

(Page numbers and line numbers in the replies are those of the revised paper)

1) Although I did not find this an exceptionally strong or groundbreaking set of studies, I also didn’t have a strong reason why the paper should be rejected from this journal. I mostly had suggestions for further clarification in various statements made in the manuscript, as listed below.

Thank you for your expert review. We revised the manuscript in accordance with your comments. Please see below for details.

2) Comments (issues that need clarification):

p. 3; line 27: What do you mean by “how to deal with physical attractiveness stereotypes”?

Corrected as follows: “Knowing that facial attractiveness is malleable and dependent on many psychological factors may provide some insights into how we can cope with appearance-related prejudices and stereotypes that may undermine psychological wellbeing.” (p. 3. line 26–28)

3) p. 3; line 28: I would add, “Perceptions of..” or “Judgements of…” before Facial attractiveness.

We added “Perceptions of” to the sentence. (p. 3, line 29)

4) p. 3; line 29: What is the meaning of “shared tastes”?

Here we used “shared tastes” to refer to the widely shared standards of facial attractiveness, such like facial symmetry and cultural standards. However, what the authors tried to emphasize here was that there are sbustantial contributions of private tastes for facial attractiveness (in addition to the shared taestes). We revised the part as follows: “Perceptions of facial attractiveness are not always based on physical features. They are influenced considerably by private tastes [34].” (p. 3, line 29–30)

5) p. 3; line 30: I would change to “factors affecting perceptions of facial attractiveness…”

Corrected as noted (p. 4, line 2).

6) p. 4; line 2: Could you say more about the meaning of “hair,” such as length or color?

Yes, the previous study [47] examined the effect of hairstyles on the facial attractiveness judgments. The term was corrected to “hairstyle.” (p. 4, line 5)

7) p. 4; line 3: You might change to “Red color was first shown to increase…”

Corrected as noted (p. 4, line 5–6).

8) p. 4; line 9: I would add “the target’s” before facial attractiveness.

Corrected as noted (p. 4, line 12).

9) p. 4; line 10: What is the meaning of a “unfairly disadvantaged target person (and “unfairly advantaged target” later in the sentence)? Also, I would add “a” and “an” before these phrases.

We corrected the part as follows: “The facial attractiveness of an unfairly disadvantaged job applicant was rated higher than that of an unfairly advantaged applicant [53]” (p. 4, line 12–14). Please see [53] for details of the experimental procedure.

10) p. 4; line 12: Can you give an example in parentheses of “Favorable behaviors”?

This sentence referred to the Nisbett and Wilson’s [55] study in the following sentence. To clarify this, we added “For example” as follows: “Favorable behaviors of a target person increase attractiveness. For example, using video stimuli, Nisbett and Wilson [55] demonstrated that the physical appearance of a target person behaving warmly and friendly was rated as more appealing than cold and distant targets.” (p. 4, line 15–18)

11) As you discuss other factors that may influence perceptions of physical attractiveness, you might also refer to prior interactions with the person, as I believe there is research to show that if there is prior interaction (even very limited), the ratings are higher.

In the revised Introduction section, we referred to some studies showing that the physical attractiveness of well-known person is rated higher than that of strangers ([35–37], p. 4, line 1–2).

12) p. 5; line 4: You might not want to refer to a “difficult-to-interpret interaction effect” unless you are to explain it.

We omitted the sentence.

13) p. 5; line 10: Can you explain more about ear length and chin shape?

Hassin & Trope [57, Study 6] presented a face image together with a verbal description (kind or mean) of the target person. As a result, the face of the kind target was rated as having rounder chin and shorter ears (see their Table 5). We revised the sentence as follows: “(e.g., a kind target was rated as having shorter ears and rounder chin [57])” (p. 5, line 16)

14) p. 5; line 16: For Experiment 2, you might also refer to the geographical location here, since you do for Experiment 1.

We revised the Participants section of Experiment 2 as follows: “The experiment was conducted in the Japanese language (thus, most participants were presumed to be residents of Japan, although we did not ask them where they lived).” (p. 10, line 13–14)

Experiment 2 participants were online workers of the Japanese crowdsourcing platform. Although the platform is operated in Japanese language, it seems possible to register as a worker from abroad.

15) p. 5 line 23: I find it a little odd that shirt attractiveness was varied in an experiment, and would like to see more justification for doing so.

Actually, the purpose of the shirt attractiveness manipulation was exploratory. In the first author’s previous study [67], it was found that the facial attractiveness influenced the ratings of shirt attractiveness. Hence we tried to examine the opposite influence (shirt attractiveness to facial attractiveness rating). This was stated in the introduction of Experiment 2 (p. 9, line 27–30).

In the revised Figure 1 caption we noted the purpose of the shirt manipulation: “Shirt attractiveness was varied in Experiment 2 to test whether it influenced on the facial attractiveness ratings (**c**).” (pp. 5–6)

16) p. 6; I’m a little confused about the pre-registered study on the issue of mood, and wondered why more information about this study was not presented in the manuscript.
This study was problemating in many points.

It was a kind of pilot study, and the failure of this study was one of our motivations for Experiment 4. The pilot study was problematic in a number of ways, so we mentioned it only briefly in the paper. Since it was rather confusing, we omit it in the revised paper.

The pilot study adopted movie clip stimuli to manipulate target honesty. The content of the movie clips was created by us anew. Therefore the materials used was quite different from those of Paunonen’s (2006). Further, the sample size was insufficient. As a result, we did not find the honesty premium effect on facial attractiveness ratings. This replication failure might be attributable to the new stimuli, the small sample size, or both. Experiment 4 was designed so that the stimuli and the procedures were as much as possible the same as in the previous study.

17) p. 7; line 5: You might refer to it as a pilot study rather than a preliminary survey.

We used “pilot study” in the revised paper throughout.

18) p. 7; line 8: You might add “in the pilot study” after “Personality descriptions were not given.”

Corrected as noted (p. 7, line 11).

19) p. 8; top: When you refer to the 20 items from Paunonen including attractiveness, you might give a couple of examples here in text.

We added some examples: “Paunonen’s 20 items were used, including attractiveness and filler items (e.g., maturity, eye roundness).” (p. 8, line 7–8)

20) p. 8; lines 4-6: I found this sentence (that begins, “Of interest was…”) to be confusing.

We revised the sentence as follows: “Paunonen found an effect of honesty not only on attractiveness but also on seven filler items (e.g., the honest target was judged to have a more feminine face and more graceful neck).” (p. 8, line 8–10)

21) p. 8; line 11: How was it possible in an online survey that two adjacent points could be marked? Or was it a paper survey?

Experiment 1 was conducted by sending a Microsoft Word document of the quiestionnaire via email. The participants reported their ratings by marking checkboxes on the document. For the 9-point scale, we made a line of 9 checkboxes. There was no automatic function to verify the responses. Probably the participants changed their mind: e.g., they first marked “2” and then marked “3” but forgot to delete the mark for “2”.

For clarity, in the revised Method section we noted that “An electronic questionnaire document (Microsoft Word file) was sent to the participants via e-mail” (p. 7, line 25)

22) p. 8; bottom: Aren’t the findings related to the effect of pre-rated facial attractiveness on facial attractiveness just a manipulation check?

Yes, testing the effect of the pre-rated attractiveness served as a manipulation check. In the revised paper we clarified this point: “rated facial attractiveness was significantly higher for targets with high pre-rated facial attractiveness (main effect of pre-rated attractiveness, *F*(1, 61) = 10.71, *p* = .002, η_p_^2^ = .149), confirming the successful manipulation” (p. 8, line 28–30)

23) p. 12: I found it a little odd that you added creativity this study with the anticipation of not finding an effect. Why not include a variable for which you expected a significant effect?

Creativity was used as a comptence-related trait. The previous studies on the effect of perceived personality on physical attractiveness judgments have examined moral-related traits (e.g., honesty, kindness) and competence-related traits (e.g., intelligence) and reported that the moral-related traits are more likely to modify facial attractiveness judgments than the competence-related traits. This framework is derived from the social psychology literature showing that the person perception is largely composed of two factors, morality/warmth and competence [69]. In the revised introduction of Experiment 3, we referred to this framework (pp. 12–13).

We conducted an additional Bayes analysis showing that the null hypothesis for the creativity effect was likely. This was reported in the revised Results section (p. 14, line 24–27): “Creativity did not affect rated attractiveness (*F*(1, 113) = 0.17, *p* = .677, η_p_^2^ = .002), nor did the 2-way interaction (*F*(1, 113) = 1.70, *p* = .195, η_p_^2^ = .015). The absence of creativity effect was supported by the moderate Bayes factor favoring the null-hypothesis (*BF*_01_ = 4.75).”

24) p. 15; line 13 and line 17: I am confused about the meaning of relevant/irrelevant, and think more information needs to be presented.

The Overview sectrion was revised as follows: “We designed four conditions based on a personality description of honesty (low/high) and face image relevance (relevant/irrelevant to the personality description). Participants read a personality description, rated the personality of the person described (manipulation check), viewed a face image of the person described (relevant) or a face image of another person (irrelevant), and rated its physical characteristics.” (p. 16, line 8–12)

25) p. 16; line 4: I am confused by this information. You state that you prepared 32 forms, and each participant was randomly assigned to one of them. But you had only 130 participants, and thus, how could you make valid group comparisons? If this truly is the case, you may need to consider adding more participants or even eliminating this study. Could this also be why the honesty effect was not replicated?

We designed four experimental conditions (high/low creativity × high/low aggressiveness), and 8 unique face images were used in all conditions. Therefores we prepared 32 (4×8) forms (see page 17, line 3–4). Please note that each participant saw only one face image. In the analysis, the 8 face images were collapsed and we contrasted the four condition.

26) Experiment 4: If mood really was of interest, why wasn’t mood a manipulated independent variable?

It is already known that experimentally manipulated moods alter facial attractiveness judgments ([41,42], see p. 6, line 9). Our purpose was not to replicate these findings. The purpose of Experiment 4 was to test whether the “honesty premium” effect was attributable to moods.

27) General Discussion: It would seem that you should discuss honesty as a “central trait” and the social psych research on how central traits can change the interpretation of other information presented simultaneously.

Thank you for the suggestion. The social psychology theories on person perception are relevant and can help us in our discussions. In the revised Experiment 3 and General Discussion, we added paragraphs on the “central traits” and the two-factor model (warmth and competence) of person perception (pp. 12, 20). We cited the classical Asch (1946) [75] and other papers [69–71].

Our experiment suggested that honesty and aggressiveness modified the perception of facial attractiveness while creativitiy did not. Paunoen (2006) [60] also showed the honesty effect and the absense of the effect of intelligence. Some other studies also reported that the warmth/morality-related traits influence the attractiveness judgments but the competence had little or no effect (e.g., [58]). This pattern of retults is consistent with the two-factor model (warmth and competence) of person perception.

28) Very minr editing suggestions:

p. 3; line 11: I would add “a” before stereotype.

p. 9; line 17: I would add “a” before “crowdsourcing platform”

p. 11; line 28: Add “the” before “target person”

p. 14; line 29: Add “the” before perceived personality” and add “a” before target person.

p. 15; line 4: Add “the” before participants.

Thank you for the suggestions. We corrected them.
