# Supplementary material for: Good conduct makes your face attractive: The effect of personality perception on facial attractiveness judgments
Source: PLoS One. 2023 Feb 13;18(2):e0281758. doi: 10.1371/journal.pone.0281758 (PMC9925008; doi:10.1371/journal.pone.0281758)
Supplement: Attachment — Submitted filename: ResponseToReviewers.docx [file pone.0281758.s008.docx]

PONE-D-22-24839R1 “Good conduct makes your face attractive: The effect of personality perception on facial attractiveness judgments”

Responses to the comments by Reviewer #2

(Page numbers and line numbers in the replies are those of the revised paper)

1) The authors have revised the manuscript in a satisfactory way and also written a reasonable cover letter indicating how they have responded to reviewer suggestions from the first round of review. Below, I identify several issues that could still use clarification in the manuscript.

Abstract; line 3: You state that human facial attractiveness is related to physical features, such as averageness and symmetry. I think some readers will have no idea of what you mean by averageness, as it can have another meaning too in regard to physical attractiveness (just being an average looking person). I would suggest you provide a different example, such as “clear complexion.”

Corrected as suggested (line 3, Abstract).

2) Abstract; line 5: When you write, “Here, we examined the effect…” add “judgments of attractiveness”.

We revised the sentence as follows: “Here, we examined the effect of the personality information of a target person on facial attractiveness judgments.” (line 5–6, Abstract).

3) Abstract; line 11: The phrase, “whereas aggressive personality of a male target might decrease the rated facial attractiveness” should be replaced with what finding you actually obtained.

We avoided definitive writing here because the statistically significant effect of aggressiveness on the rated attractiveness for male faces was found in the ad hoc analysis where the achieved statistical power was not large enough (see p. 15, “Effect of targe gender” section). We revised the part as follows: “while an aggressive personality was suggested to decrease the rated facial attractiveness of male targets.” (line 11–12, Abstract)

4) p. 3; line 12: I found it confusing (and a little annoying actually) when you have a sentence such as “It is noteworthy that the validity of such a stereotype has been questioned (but see…). First, you don’t say how the validity has been questioned, and then you don’t give us an idea of what the “but see” would say. So, both parts of the sentence seem misleading, and particularly in combination.

This part was revised as follows: “It is noteworthy that the validity of such a stereotype has been questioned, as physical attractiveness does not reliably predict actual competence or personality [5,9,10], although some studies suggest a partial relationship between facial appearance and actual personality [11].” (p. 3, line 11–13)

5) p. 3; line 20; ditto the two “but see”.

We revised the part to avoid using “but see.” We believe that it is important for science (and even helpful for readers) to presenting conflicting findings without ignoring them.

“Indeed, studies have sought objective determinants of facial attractiveness, such as the size of facial parts [25], symmetry [26,27], and averageness [28], although debate about these factors persist [29,30].” (p. 3, line 20–22)

6) p. 3; line 24 and 25: Judgments of attractiveness are not the same as “being attracted”, but this sentence implies they are the same.

We do not intend to imply that. Here we used the term “attractiveness” (a psychological construct), not “attractiveness judgments” (observable behaviors). To clarify this point, we revised the sentence as follows: “However, it seems quite important to remember that attractiveness is a psychological construct that represents an observer’s internal perception of being attracted, not an objective entity” (p. 3, line 26–27)

7) p. 3; line 25: You state that attractiveness is not an objective reality and that beauty is in the eye of the beholder. However, earlier in the same page (line 17), you had indicated that visual perception of facial attractiveness is consistent among observers.

The consistency of attractiveness judgments is not 100%. In human behavior of any kind, there is always both consistency among people and idiosyncrasy. The presence of consistency in attractiveness judgments does not mean that there is no room for individual differences. We do not insist that there is no effect of physical features on facial attractiveness judgments. As we wrote in the abstract, we concluded that “facial attractiveness is malleable and dependent on various *physical and non-physical* information.” We focused on non-physical factors in this paper because they have received little attention in the study of facial attractiveness.

To clarify this point, we added “in addition to physical ones” to the sentence of p. 3; “Knowing that facial attractiveness is malleable and dependent on many psychological factors in addition to physical ones may provide some insights into how we can cope with appearance-related prejudices and stereotypes that may undermine psychological wellbeing” (p. 3, line 28–30).

8) p. 3; line 28: Can you provide a citation at the end of this sentence saying that appearance-related prejudice and stereotypes may undermine psychological wellbeing?

We added a reference to [1]. (p. 4, line 1)

9) p. 3; line 30: I’m still not sure what you mean by “private tastes”. I think you could choose a different wording.

The term “private taste” was from the title of the previous study [34]. We had adhered to the original wording. In the revised manuscript we replaced “private taste” with “idiosyncratic standards of beauty” (p. 4, line 3). For details, please see [34].

10) p. 4; line 13: I think you give enough other examples that it would be fine to delete the example of the study referring to an “unfairly disadvantaged job applicant” and an “unfairly advantaged applicant”. I think that is just a confusing example.

We dropped the sentence. In the revised paper, we only briefly referred to the study: “Situational context [53], attitude similarity [54] and in-group bias [38] are also known to influence facial attractiveness judgments” (p. 4, line 15–17). For details of the “unfairly disadvantaged job applicant”, please see the study [53].

11) p. 5; line 5: Maybe change to: a personality description that included the manipulations of honesty, intelligence, and independence.

Changed as suggested (p. 5, line 6)

12) p. 7; top; Explain more where the participants were obtained from and how they were recruited.

In the revised paper we explained as follows: “They were recruited through electronic flyers distributed after classes and mail-based snowball sampling.” (p. 7, line 5–6)

13) p. 7: Because you had both male and female targets (and described that at the top of p. 7), it is confusing when you don’t refer to the gender of the target at the bottom of the page (that it is just ignored).

We repeated the issue of target gender in the revised Procedure section: “We created 16 versions of the form in total (two levels of honesty × eight face images [4 male, 4 female]).” (p. 7, line 29–30)

14) p. 8; line 24: You state that “Pre-rated facial attractiveness had almost no effect…” That sentence seems too ambiguous. Either there are effects or there are none.

There were one significant effect and six non-significant effects. Among the seven personality rating items, only one item (dependent–independent) was significantly modified by pre-rated facial attractiveness (*p* = .015), while other items were not. We reported these statistical results in S1 Table of the Supporting Information. In the revised paper, we added a reference to S1 Table (p. 8, line 27).

15) p. 9; Summary: In this brief Summary, summarize again the findings that lead to the conclusion that there was an “honesty premium” effect.

We added the following summary: “Prior presentation of verbal information about honest personality increased the rated attractiveness of subsequently presented target faces.” (p. 9, line 20–21)

16) p. 9; bottom: I’m still confused why clothing was manipulated rather than facial attractiveness.

As we wrote in the previous reply letter, it was an exploratory attempt without clear hypotheses. In the revised paper we explicitly stated that it was exploratory: “Another purpose of Experiment 2 was exploratory, to test whether attractive clothing influenced the judgments of facial attractiveness.” (p. 10, line 3)

17) p. 10; line 19: Provide another statement about what the two attractiveness t-shirts looked like and what the unattractive t-shirts looked like.

Figure 3c shows the examples of the attractive and unattractive t-shirts. In the revised paper we repeated a reference to Figure 3c (p. 10, line 24).

18) p. 12; top: Were there any significant interactions?

There were two significant interactions for the physical rating items other than attractiveness; 1) honesty×target gender interaction effect on “poor health”—“good health” rating, *p* = .001; 2) the four-way interaction effect on “coarse hair”—“fine hair” rating, *p* = .040. We omitted them because they seemed trivial for the purpose of our study.

19) p. 12; line 20: You might consider adding this phrase in parentheses after “attractiveness ratings because aggressiveness (or its opposite)…

We suppose that the reviewer referred to p. 12, line 29 (p. 13, line 4 in the second revised manuscript). We kept the original sentence because in this paper “aggressiveness” refers to the personality dimension, not an aggressive personality. We would like to keep the consistent terminology.

20) p. 13; Participants: Where did the participants come from and how were they recruited?

We added the following sentence: “As in Experiment 1, we recruited the participants via electronic flyers and emails.” (p. 13, line 17–18)

21) p. 16: Ditto the question for Experiment 4.

The revised Participants section described the method as follows: “Eighty-two undergraduate students completed the experiment in exchange for course credit. An additional 49 undergraduates participated and received a voucher reward of 300 JPY. They were recruited through electronic flyers and emails.” (p. 16, line 28–29)
